# Highly Acidic Conditions Drastically Alter the Chemical Composition and Absorption Coefficient of α-Pinene Secondary Organic Aerosol

**DOI:** 10.1021/acsearthspacechem.2c00249

**Published:** 2022-11-22

**Authors:** Cynthia Wong, Sijia Liu, Sergey A. Nizkorodov

**Affiliations:** Department of Chemistry, University of California, Irvine, California 92697-2025, United States

**Keywords:** particulate matter, organic aerosol, acid-catalyzed reactions, chemical aging, organosulfur compounds, brown carbon, light-absorbing aerosol, aerosol fluorescence

## Abstract

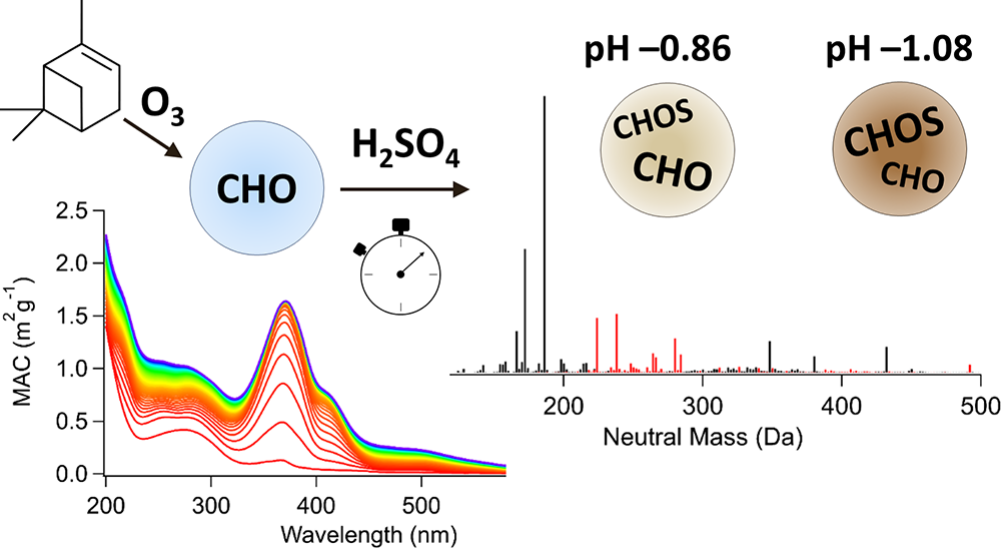

Secondary organic aerosols (SOA), formed through the gas-phase oxidation of volatile organic compounds (VOCs), can reside in the atmosphere for many days. The formation of SOA takes place rapidly within hours after VOC emissions, but SOA can undergo much slower physical and chemical processes throughout their lifetime in the atmosphere. The acidity of atmospheric aerosols spans a wide range, with the most acidic particles having negative pH values, which can promote acid-catalyzed reactions. The goal of this work is to elucidate poorly understood mechanisms and rates of acid-catalyzed aging of mixtures of representative SOA compounds. SOA were generated by the ozonolysis of α-pinene in a continuous flow reactor and then collected using a foil substrate. SOA samples were extracted and aged by exposure to varying concentrations of aqueous H_2_SO_4_ for 1–2 days. Chemical analysis of fresh and aged samples was conducted using ultra-performance liquid chromatography coupled with photodiode array spectrophotomety and high-resolution mass spectrometry. In addition, UV–vis spectrophotometry and fluorescence spectrophotometry were used to examine the changes in optical properties before and after aging. We observed that SOA that aged in moderately acidic conditions (pH from 0 to 4) experienced small changes in composition, while SOA that aged in a highly acidic environment (pH from −1 to 0) experienced more dramatic changes in composition, including the formation of compounds containing sulfur. Additionally, at highly acidic conditions, light-absorbing and fluorescent compounds appeared, but their identities could not be ascertained due to their small relative abundance. This study shows that acidity is a major driver of SOA aging, resulting in a large change in the chemical composition and optical properties of aerosols in regions where high concentrations of H_2_SO_4_ persist, such as upper troposphere and lower stratosphere.

## Introduction

Aerosols play an important role in the atmosphere directly by absorbing and scattering radiant energy and indirectly by acting as cloud condensation nuclei and ice nucleating particles.^1^ They can contribute to poor air quality, causing decreased visibility and adverse health effects.^2,3^ Organic aerosols are ubiquitous and account for the dominant fraction of aerosols in the atmosphere.^4^ Secondary organic aerosols (SOA), which are primarily formed through either nucleation, condensation, or multiphase chemical processing of oxidation products of volatile organic compounds (VOCs), have highly complex composition and a wide range of physical and chemical characteristics. The lifetimes of SOA can be as long as several days or even weeks, with the SOA in the upper troposphere having longer lifetimes because the removal mechanisms for particles at this altitude are inefficient.^5^

The acidity of atmospheric aqueous phase (i.e., aerosol particles, cloud droplets, and fog droplets) is an important factor that can influence physical and chemical processes. There is a wide range of acidity in the atmosphere. Cloud and fog droplets can have pH values ranging from +2 to +7, while aerosol particles tend to be more acidic and have a wider range, with pH values from −1 to +8, depending on their source, chemical composition, and ambient relative humidity.^6,7^

Organic reactions in the atmosphere can be either acid-catalyzed or acid-driven, the difference being that in the latter the protons from the acid are incorporated into the products formed. Hemiacetal and acetal formation are dependent on acidic conditions in which a compound containing a hydroxyl group is added to a carbonyl compound after the protonation of the carbonyl group, followed by the addition of an alcohol. This reaction has been shown to be significant for aqueous SOA formation from carbonyl compounds such as glyoxal and methylglyoxal.^8−10^ The rates of hydration of complex aldehydes, ketones, and carbonyls have been shown to be strongly influenced by the presence of acids once partitioned into the aqueous phase in which the carbonyl group is converted into gem-diols.^11−13^ Aldol condensation can be highly pH-sensitive due to the acid-catalyzed nature of the enol formation and the role of the protonated carbonyl. Several studies of atmospherically relevant carbonyls have reported that this reaction is favorable under strongly acidic pH < 2,^14−18^ while another study reported that this aldol condensation can occur at pH as high as +4 to +5.^19^ Multiple studies have shown that the rate of esterification will increase with increasing acidity because the carboxyl group needs to be protonated to form a carbocation before a nucleophilic attack by an alcohol.^6,20−22^ Finally, acids can also facilitate nucleophilic addition through isoprene and monoterpene epoxide protonation, which can lead to organosulfate formation.^23−26^

The effects of seed particle acidity on the growth of SOA have been extensively investigated. Chamber studies have shown enhanced production of SOA in the presence of acidic seed particles, suggesting the importance of acid-catalyzed processes.^27−36^ The presence of acidic sulfate seeds in the formation of SOA from various combinations of VOC precursors (i.e., isoprene, α-pinene, d-limonene, *m*-xylene, toluene, benzene, etc.) and oxidants (i.e., O_3_ and OH) can probe changes in organic aerosol chemical properties such as mass yields, oxidation state, and composition, including the formation of larger oligomers, organosulfates, and light-absorbing compounds.^27−36^ Studies have shown that the reactive uptake was observed for various individual products of oxidation of biogenic VOCs onto acidified particles. For example, pinonaldehyde uptake on inorganic sulfate seed aerosols resulted in oligomer and organosulfate formation.^15,37^ The uptake of isoprene-derived epoxydiols in solutions of sulfates [i.e., H_2_SO_4_, Na_2_SO_4_, (NH_4_)_2_SO_4_, and (NH_4_)HSO_4_] leads to the formation of polyols and sulfate esters as well as light-absorbing compounds, with the rate of formation being dependent on the acidity of the solution.^25,38,39^ Additionally, previous work has also focused on the reactive uptake of aldehydes and ketones into sulfuric acid solutions and particles, mimicking stratospheric aerosols.^14,16,17,40,41^ It was found that the rate constant was dependent on the chain length and acidity, and acidity also facilitated the formation of light-absorbing compounds and oligomers.^14,16,17,40,42^

While the effects of acidity on the initial SOA formation is well understood, less is known about the role of acids in the chemical aging of organic aerosols occurring over longer timescales. A previous work in our group found that the evaporation of bulk biogenic and anthropogenic SOA solutions in the presence of sulfuric acid enhanced the absorbance at visible wavelengths and resulted in significant changes in the chemical composition, including organosulfate formation.^41,43^ However, the pH was difficult to be measured during evaporation, and it was not clear at what specific pH values these changes took place. Additionally, other studies were conducted either on a limited range of acidity, limited number of organic compounds, or short timescale. We hypothesize that aging SOA in acid for an extended period of time (days) would probe acid-catalyzed and acid-driven chemical reactions, leading to significant changes in the composition. The goal of this work is to elucidate the mechanisms and rates of aging processes of mixtures of representative SOA compounds in the presence of increasing levels of acidity. Our results suggest that SOA aging processes are accelerated in sulfuric acid solutions at atmospherically relevant pH, with organosulfates and light-absorbing products formed under highly acidic conditions.

## Experimental Methods

### SOA Generation

SOA samples were generated in a ∼20 L continuous flow reactor under dry and dark conditions. The reactor was purged with zero air (Parker 75-62 purge gas generator) prior to each experiment, and ozone was introduced into the reactor by flowing pure oxygen through a commercial ozone generator (OzoneTech OZ2SS-SS) at 0.5 slm (standard liters per minute). Liquid α-pinene (Fisher Scientific, 98% purity) was injected into ∼5 slm flow of zero air using a syringe pump at a constant rate of ∼25 μL h^–1^. The estimated initial mixing ratios of ozone and VOC were 14 and 10 ppm, respectively. This high loading was used to generate enough SOA material for the analysis described below, which favors RO_2_ + RO_2_ reactions and prompts higher volatile compounds to partition in the particles. The SOA compounds that are generated through this method are still observed in field studies; however, future studies should focus on reducing the loading to increase the relevance of the work.

SOA passed through a 1 m charcoal denuder to remove ozone and reduce the concentration of VOCs and was collected onto a foil substrate on stage 7 (0.32–0.56 μm) of a micro-orifice uniform deposit impactor (MOUDI, model 110R) at a flow rate of ∼30 slm to create a uniform deposition of particles on the substrate (with 5.5 slm coming from the cell and the rest coming from a filtered lab air). The reduction in VOC concentrations in the denuded flow, combined with dilution in MOUDI, is expected to remove the more volatile compounds from particles and thus preferentially collect the majority of low-volatility compounds in the SOA, which are more atmospherically relevant. The mass on the foil substrate was typically ∼2 mg. Some samples were vacuum-sealed and frozen, while other samples were aged in acidic conditions until mass spectrometry (MS) analysis was performed.

### Aging in Sulfuric Acid

Each SOA substrate was cut into three approximately equal segments. SOA from the first segment was extracted and then aged in a solution of sulfuric acid to monitor the change in chemical composition by MS; SOA from the second segment was similarly aged in a sulfuric acid solution to detect light-absorbing compounds by spectrophotometry; and finally, the last segment was sealed with a food-sealer and frozen for control MS experiments. The protocol used for these experiments is illustrated in Figure S1. Two segments containing SOA were first extracted using ∼4 mL of acetonitrile into a scintillation vial by shaking it for ∼10 min. The solvent was then removed from the vial using a rotary evaporator at ∼25 °C. This process may have removed semivolatile compounds that have partitioned into the particles; however, we were mostly concerned about retaining low-volatility compounds in the SOA as they are more atmospherically relevant. A volume of ∼4 mL of a dilute aqueous solution of sulfuric acid (Fisher Scientific, 96% purity), prepared as described in Table 1, was added to the vials, resulting in a mass concentration of 180–200 μg/mL. Note that the dilute aqueous solutions of sulfuric acid were prepared in advance and cooled to room temperature before using them to extract SOA (in no experiments concentrated sulfuric acid was added to SOA solutions because this would produce a temperature increase). When appropriate (pH > 0), a pH meter (Mettler Toledo SevenEasy S20) was used to determine the acidity of the SOA and sulfuric acid solution. One sample was immediately placed in the spectrophotometer (Shimadzu UV-2450) to monitor the absorption spectrum as a function of time, and the other solution was left undisturbed in darkness for 2 days until MS analysis. The SOA on the frozen control was extracted immediately before the analysis using the same method as described above; however, ∼4 mL of nanopure water was added to the scintillation vial as the solvent.

**Table 1:** Solution Acidity in Aging Experiments^a^

estimated concentration of H_2_SO_4_	pH meter reading	[HSO_4_^–^] (mol/kg)	[SO_4_^2–^] (mol/kg)	[H^+^] (mol/kg)	activity coefficient of H^+^	effective pH = −log[H^+^]
0 M	4.3					control
0.52 mM	2.7	0.0000386	0.000481	0.00100	0.96	3.00
6.4 mM	1.9	0.00240	0.00402	0.0105	0.88	1.98
90 mM	1.1	0.0654	0.0249	0.115	0.76	0.94
1.0 M		0.754	0.249	1.25	0.78	–0.01
1.8 M		1.26	0.508	2.28	0.92	–0.36
3.2 M		2.15	1.01	4.17	1.4	–0.62
5.6 M		3.96	1.67	7.30	3.7	–0.86
10 M		8.10	1.93	12.0	20	–1.08

a The first column lists the molar concentration of H_2_SO_4_ added to the solution. The molalities of [H^+^] and other ions were calculated using the E-AIM (http://www.aim.env.uea.ac.uk/aim/aim.php). The effective pH values listed in the last column and quoted in the paper represent the negative logarithm of molality of H^+^.

### Measurements and Modeling of pH

A pH meter (Mettler Toledo SevenEasy S20) was used to determine the acidity of the sulfuric acid solution, the SOA, and sulfuric acid sample (pH > 0). However, determining the pH for highly acidic (pH < 0) conditions is difficult due to the limited range of the pH probe (pH 0–14). Therefore, extended aerosol inorganic model I (E-AIM) was utilized to estimate the pH of the sulfuric acid solution.^44−46^ By inputting the starting moles of H^+^ and SO_4_^2–^ present and ambient conditions of the solution, the aqueous molality and mole fractions of H^+^ can be determined and used to calculate the effective pH of the solution
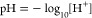
1where [H^+^] is the molality of protons. The resulting effective pH values ranged from −1 to +3, with the exact values for each trial listed in Table 1. It is noted that for the positive pH values, the pH meter readings and the E-AIM output agreed reasonably well, with small deviations. The E-AIM model also provided activity coefficients for H^+^, which are included in Table 1 and can be used to calculate the pH corrected for the activity. For the remainder of this paper, we will refer to the solutions by their effective pH calculated from eq 1.

### MS Analysis

The analysis of fresh and aged samples was conducted using a Thermo Scientific Vanquish Horizon ultra-performance liquid chromatography (UPLC) system coupled with a Vanquish Horizon photodiode array (PDA) spectrophotometer and a Thermo Scientific Q Exactive Plus Orbitrap high-resolution mass spectrometer to examine the chemical composition of SOA before and after aging. UPLC separation was carried out on a Phenomenex Luna Omega Polar C18 column, 150 × 2.1 mm, with 1.6 μm particles and 100 Å pores, with the temperature set to 30 °C at a flow rate of 0.3 mL/min. The mobile phase consisted of water (eluent A) and acetonitrile (eluent B), each containing 0.1% formic acid. The gradient elution was programmed as follows: 0–3 min 95% eluent A; 3–14 min linear ramp to 95% eluent B; 14–16 min hold at 95% eluent B; 16–22 min return to 95% eluent A. The mass spectrometer was operated with a spray voltage of 2.5 kV and a resolving power of *m*/Δ*m* = 1.4 × 10^5^. Most of the analysis was done with negative ion mode data to observe the presence of organosulfur compounds; however, positive ion mode data sets were also acquired for SOA aged at pH −1.08 to analyze potential chromophores.

Peaks were imported to Thermo Scientific program FreeStyle 1.6 and integrated between 2 and 16 min, which correspond to the elution of the majority of SOA compounds in the chromatogram. The peak *m*/*z* values and abundances were extracted from the raw mass spectra using the Decon2LS program (https://omics.pnl.gov/software/decontools-decon2ls) and then processed with the in-house software. The peaks corresponding to molecules with ^13^C atoms or obvious impurities and ion fragments, signified by anomalous full width at half-maximum and mass defects, were removed from the peak table. Additionally, peaks with a solvent/sample ratio of more than 1 were considered impurities and were excluded from further analysis. The solvent peak abundances were then subtracted from the peak abundances in the samples. The peaks were assigned to formulas in two stages, first to internally calibrate the *m*/*z* axis with respect to the expected peaks for α-pinene ozonolysis and then to reassign after a minor (<0.001 *m*/*z* units) adjustment to the *m*/*z* values. The mass spectra from different samples were clustered by the molecular formulas of the neutral compounds CHO and CHOS for fresh and aged SOA, respectively. Deprotonation was assumed to be the main ionization mechanism for negative ion mass spectra.

### Spectroscopic Measurements

A UV–vis spectrophotometer (Shimadzu UV-2450) was used to monitor the formation of light-absorbing compounds over time. The spectrophotometer was programmed to collect the spectrum every 15 min for 24 h, and one more spectrum was then collected at the 48 h time point. Mass absorption coefficients (MACs), in units of cm^2^ g^–1^, were calculated using eq 2, where *A*_10_ is the base-10 absorbance, *C*_mass_ (g cm^–3^) is the concentration of SOA in solution which was estimated by measuring the mass of the foil segment before and after extraction, and *b* (cm) is the path length (standard 10 mm cuvettes were used in these experiments)
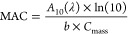
2

Aliquots of some samples were also examined using a Cary Eclipse fluorescence spectrometer to investigate the presence of fluorescent molecules. The parameters used for these experiments mimic those of the past studies by our group.^47^ The background for the fluorescence spectrum was deionized water, and the samples analyzed were the SOA aged in H_2_SO_4_ after 2 days. The excitation wavelength varied over the 200–500 nm range in 5 nm steps, and the emitted fluorescence was recorded over the 300–600 nm range in 2 nm steps for the excitation–emission spectra.

## Results and Discussion

### Chemical Composition of Fresh α-Pinene SOA

Figure S2 shows a representative high-resolution mass spectrum of the fresh α-pinene ozonolysis SOA, where the peaks are normalized to the sum of peak abundances. The mass spectrum is similar to the previous ESI mass spectra reported for α-pinene ozonolysis SOA with distinct monomer (<250 Da) and dimer (250–450 Da) regions, which correspond to products with one and two oxygenated α-pinene units. The five most abundant monomer peaks have monoisotopic molecular masses of 186.089 Da (C_9_H_14_O_4_, pinic acid), 172.074 Da (C_8_H_12_O_4_, terpenylic acid), 198.089 Da (C_10_H_14_O_4_, oxopinonic acid), 200.105 Da (C_10_H_16_O_4_, 10-hydroxypinonic acid), and 216.100 Da (C_10_H_16_O_5_). The molecular formulas and most likely identities for these products appear in parentheses.^48,49^ The five most abundant dimer peaks have monoisotopic molecular masses of 354.204 Da (C_19_H_30_O_6_), 368.184 Da (C_19_H_28_O_7_), 338.209 Da (C_19_H_30_O_5_), 358.163 Da (C_17_H_26_O_8_), and 370.199 Da (C_19_H_30_O_7_). These most abundant monomers and dimers have been commonly observed in both lab-generated and ambient SOA samples, confirming that, despite the high mass loadings in the flow tube reactor, our starting SOA material is representative of the typical α-pinene SOA.^49−53^

### Chemical Composition of Aged α-Pinene SOA

**Figure 1:**
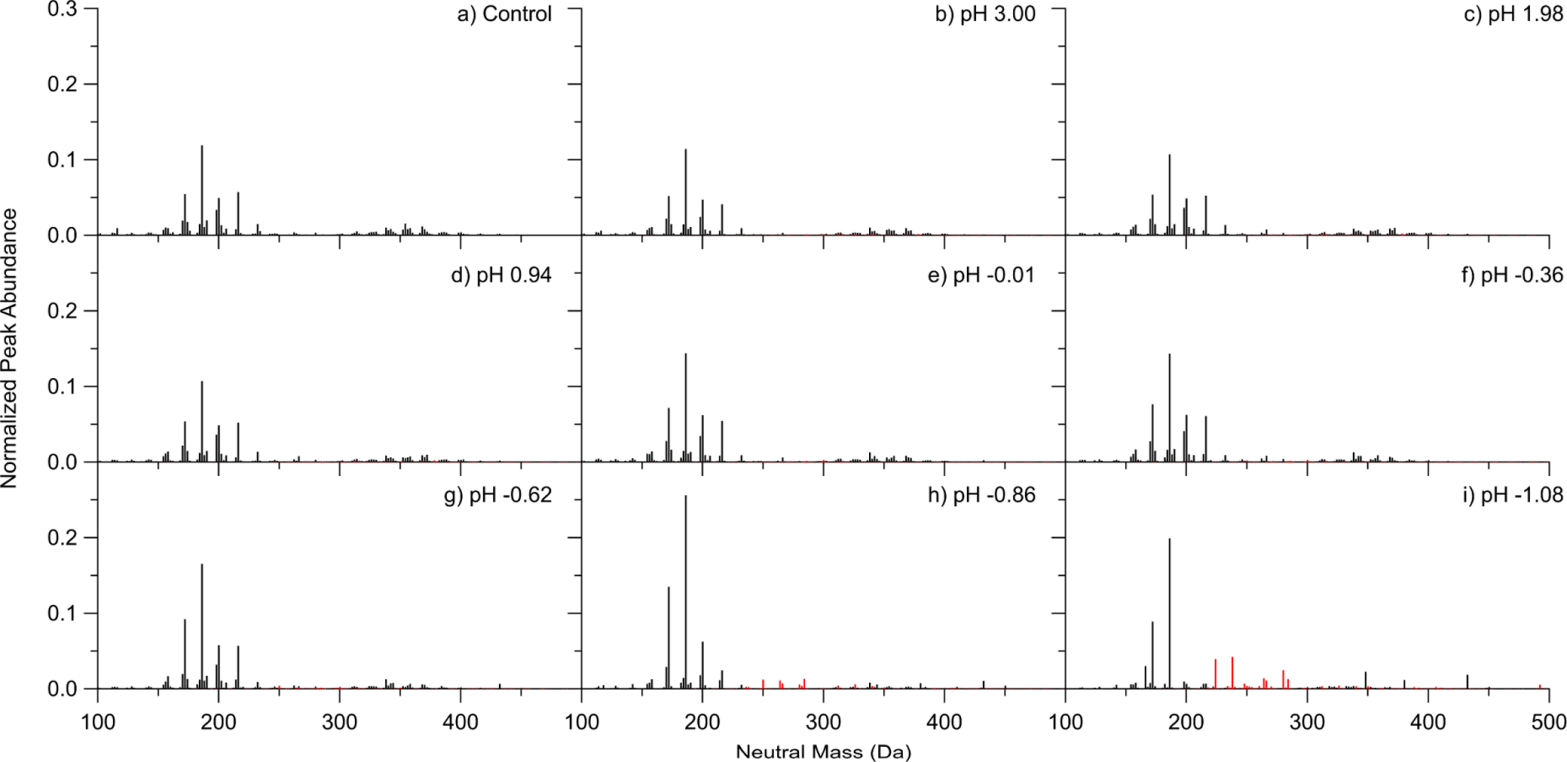
Mass spectra of α-pinene ozonolysis SOA samples aged for 2 days in (a) 0 M (control), (b) 0.52 mM (pH 3.00), (c) 6.4 mM (pH 1.98), (d) 90 mM (pH 0.94), (e) 1.0 M (pH −0.01), (f) 1.8 M (pH −0.36), (g) 3.2 M (pH −0.62), (h) 5.6 M (pH −0.86), and (i) 10 M (pH −1.08) of H_2_SO_4_. Black traces correspond to CHO compounds, while red traces correspond to CHOS compounds. Mass spectra were derived by integrating over 2–16 min for each of the LC run and normalizing by the combined peak abundance.

Figure 1 shows the mass spectra of α-pinene ozonolysis SOA samples aged for 2 days in various concentrations of sulfuric acid. Specific details of the aging conditions are outlined in Table 1. The mass spectra of the SOA aged in moderately acidic conditions (Figure 1a–c) are very similar to the mass spectrum of fresh SOA (Figure S2). The mass spectra have similar well-defined monomer (<250 Da) and dimer regions (250–450 Da). The dominant peaks in the monomer and dimer regions are also similar. The top five monomer peaks correspond to 186.089 Da (C_9_H_14_O_4_, pinic acid), 172.074 Da (C_8_H_12_O_4_, terpenylic acid), 198.089 Da (C_10_H_14_O_4_, oxopinonic acid), 200.105 Da (C_10_H_16_O_4_, 10-hydroxypinonic acid), and 216.100 Da (C_10_H_16_O_5_). The most prominent dimer peaks correspond to 338.2093 Da (C_19_H_30_O_5_) and 368.184 Da (C_19_H_28_O_7_). Additionally, the peaks of organosulfur compounds are small in terms of both number and abundance. This implies little to no evidence of acid-catalyzed aging processes occurring under these moderately acidic conditions. This observation is consistent with previous works that showed that pre-existing seeds and aerosol acidity did not result in significant increases in the intensities of oligomers, likely due to the relatively low acidity of the seeds.^49,54^

In stark contrast, the mass spectrum of the SOA aged under the most acidic conditions of pH −1.08 (Figure 1i) is notably different. There is no distinct monomer or dimer region, and organosulfur compounds have prominent peak abundances. The top five peaks corresponding to CHO compounds are 186.089 Da (C_9_H_14_O_4_, pinic acid), 172.074 Da (C_8_H_12_O_4_, terpenylic acid), 166.099 Da (C_10_H_14_O_2_), 198.089 Da (C_10_H_14_O_4_), and 158.058 Da (C_7_H_10_O_4_). Additionally, the five strongest peaks corresponding to CHOS compounds are 238.051 (C_8_H_14_SO_6_), 224.036 (C_7_H_12_SO_6_), 280.062 (C_10_H_16_SO_7_), 264.067 (C_10_H_16_SO_6_), and 284.093 Da (C_10_H_20_SO_7_). These compounds have been detected in both experimental studies^36,55−57^ and field studies all over the world, including the southeast US, Denmark, China, Finland, Germany, Greenland, and Norway.^58−67^ A more detailed list of peaks of interest can be found in Table 2.

**Table 2:**
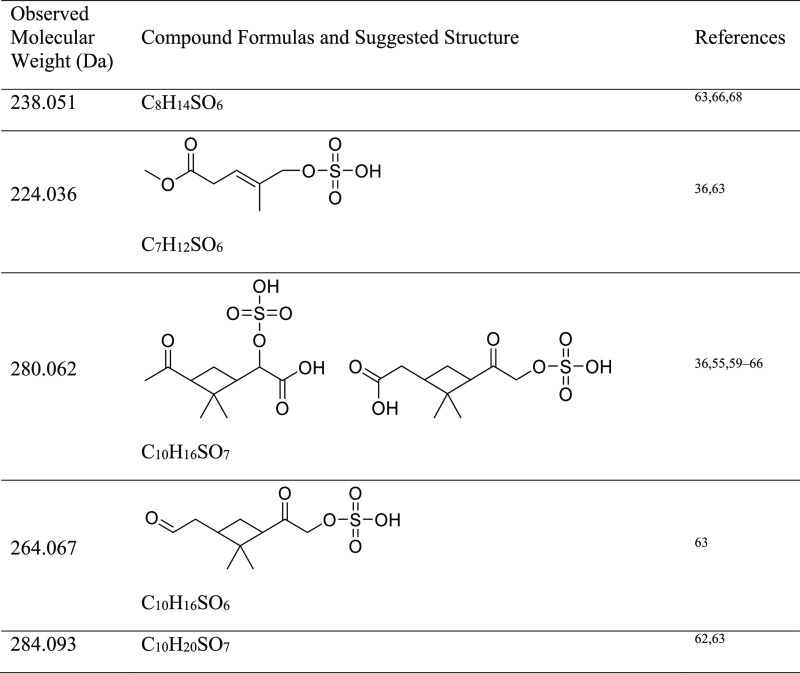
Some of the Major CHOS Compounds Detected in Aged APIN SOA at pH −1.08 by MS

Most of our experiments in Figure 1 were performed for pH between 0 and −1 to determine whether the extent of chemical change in SOA was gradual or abrupt as a function of pH. The mass spectra of SOA aged at pH 1 (Figure 1d), pH −0.01 (Figure 1e), pH −0.36 (Figure 1f), and pH −0.62 (Figure 1g) are still fairly similar to that of fresh SOA, in that the mass spectra have similar peak patterns in the monomer (<250 Da) and dimer (250–450 Da) regions. However, the mass spectrum of SOA aged at pH −0.86 (Figure 1h) has many notable differences in comparison to the fresh SOA and resembles the pH −1.08 mass spectrum (Figure 1i). These differences include changes in the peak pattern in the monomer region (<250 Da) as well as evidence in the peaks of organosulfur compounds colored in red in Figure 1. The top five monomer peaks of SOA samples aged at pH −0.86 include 186.089 Da (C_9_H_14_O_4_, pinic acid), 172.074 Da (C_8_H_12_O_4_, terpenylic acid), 200.105 Da (C_10_H_16_O_4_, 10-hydroxypinonic acid), 170.093 Da (C_9_H_14_O_3_, pinalic acid), and 216.100 Da (C_10_H_16_O_5_). Some of these peaks are the same as the strongest peaks in the fresh SOA; however, the peaks vary in intensity compared to the fresh SOA. The major CHOS compounds correspond to 284.093 (C_10_H_20_SO_7_), 250.087 (C_10_H_18_SO_5_), 264.067 (C_10_H_16_SO_6_), 266.082 (C_10_H_18_SO_6_), and 326.019 (C_18_H_30_SO_3_). In comparison to the SOA aged at pH −1.08 mass spectra, the pH −0.86 mass spectra have some key differences. These discrepancies include larger intensities of 200.105 Da (C_10_H_16_O_4_, 10-hydroxypinonic acid), 170.093 Da (C_9_H_14_O_3_, pinalic acid), and 216.100 Da (C_10_H_16_O_5_), missing some larger CHOS compounds [e.g., 238.051 (C_8_H_14_SO_6_) and 224.036 (C_7_H_12_SO_6_)]. This implies that even a relatively small change in pH (by 0.25 units) can have a strong effect on the disappearance of specific CHO and formation of CHOS compounds.

The mass spectrometric analysis suggests that aging aerosols in highly concentrated sulfuric acid leads to significant changes in chemical composition, for example, the formation of organosulfur compounds. This is further illustrated by Figure S3, which shows the relative intensity of CHO and CHOS compounds for each sample. As the pH decreases, there is an increase in the relative abundance of organosulfur compounds, especially at the lowest pH values probed here. Specifically, when α-pinene ozonolysis SOA samples were aged at pH −0.86 and pH −1.08 conditions, the fractions of the observed organosulfur compounds were ∼12 and ∼30%, respectively. The concentration of the sulfate anions increases along with acidity, and this may also contribute to the increased formation of organosulfates. However, the extent of aging increases rapidly over a narrow pH window, in which the excess sulfate concentration changes more slowly than acidity, suggesting that pH is a more important factor than sulfate concentration.

### Optical Properties and Kinetics of Aged α-Pinene SOA

**Figure 2:**
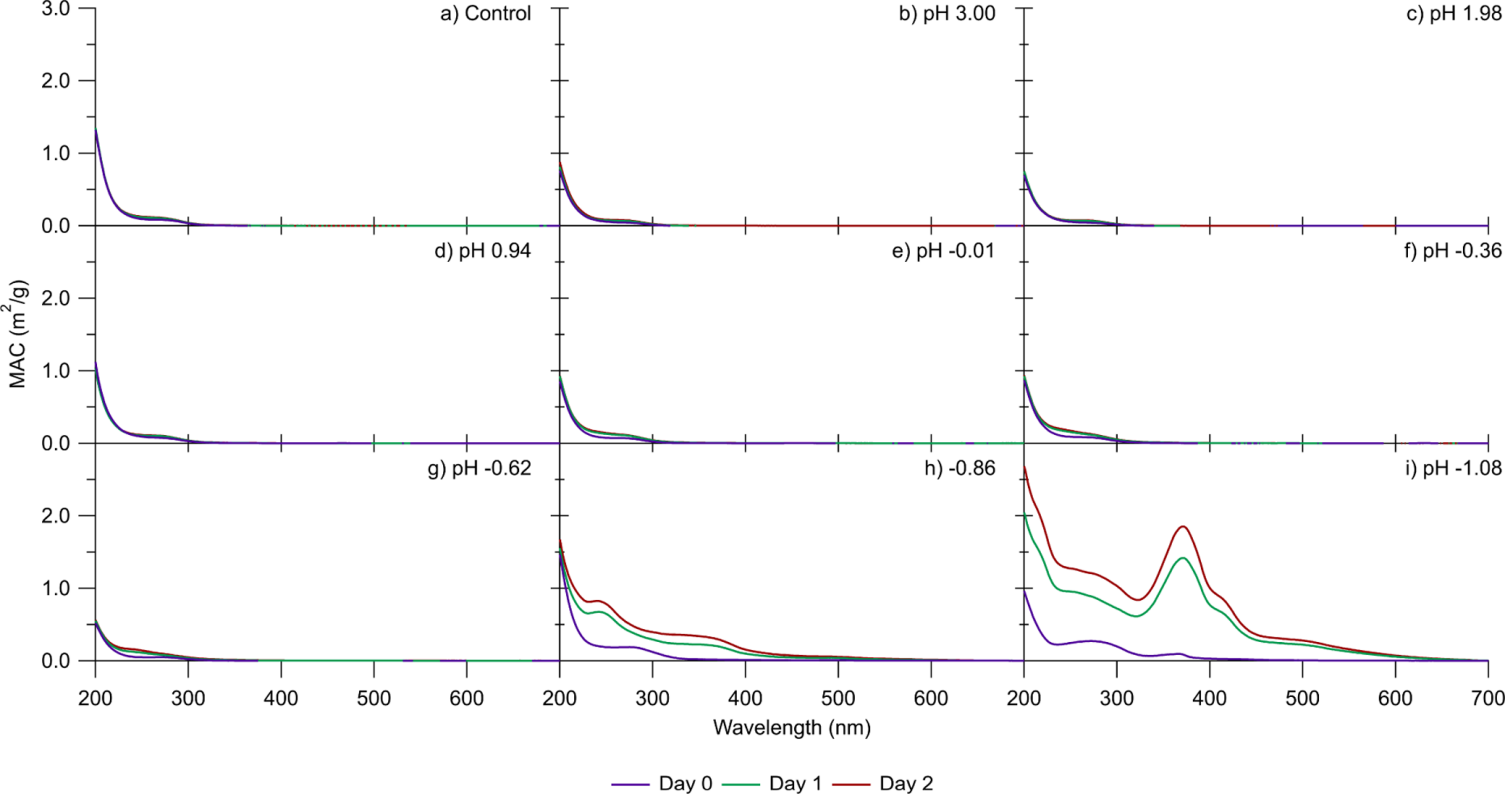
MAC spectra of α-pinene ozonolysis SOA samples aged for 0, 1, and 2 days in (a) 0 M (control), (b) 5.2 × 10^–4^ M (pH 3.00), (c) 6.4 × 10^–3^ M (pH 1.98), (d) 9.0 × 10^–2^ M (pH 0.94), (e) 1.0 M (pH −0.01), (f) 1.8 M (pH −0.36), (g) 3.2 M (pH −0.62), (h) 5.6 M (pH −0.86), and (i) 10 M (pH −1.08) solutions of H_2_SO_4_.

Wavelength-dependent MACs for aged α-pinene SOA at various concentrations of sulfuric acid at three different time points of aging are summarized in Figure 2. The details of the aging conditions used for these experiments are explained in Table 1. The MAC spectra of the SOA aged in moderately acidic conditions (Figure 2a–c) are very similar to the absorption spectra collected in previous studies.^47,69,70^ The shapes of the observed spectra of unaged SOA are consistent with the weak *n* → π* transition in carbonyls superimposed on the smooth absorption band from peroxides.^71^ Both peroxide and carbonyl functional groups are common in α-pinene ozonolysis SOA.^48,49,72^

As the concentration of sulfuric acid increases into higher acidities, the shape of the spectra does not change until the pH reaches −0.86 and −1.08. At pH −0.86, two dominant peaks appear at 239 and 351 nm after 1 day of aging, which can continue to increase during the second day of aging. The MAC spectrum of SOA aged at pH −1.08 (Figure 2f) at day 0 has a different shape from the MAC spectrum of unacidified SOA, with the small peaks at 370 and 250 nm indicating that some chemistry happens already during 1–2 min between mixing the solution and taking the first spectrum. The spectra at day 1 and day 2 have three dominant peaks present at 254, 370, and 418 nm.

There have been reports of acid-catalyzed formation of chromophores from monoterpene ozonolysis SOA.^33,41,43^ Evaporation of SOA solutions in the presence of sulfuric acid was found to enhance the absorbance, with the largest effect observed for d-limonene ozonolysis SOA. The authors attributed this observation to the acid-catalyzed dehydration resulting in higher unsaturation (e.g., as a result of aldol condensation).^41,43^ Song et al. did not observe brown carbon formation for the α-pinene O_3_ system in the presence of neutral or acidic seeds; therefore, it is likely that the pH of these seeds was not as acidic as that of the samples used in this study.^33^

**Figure 3:**
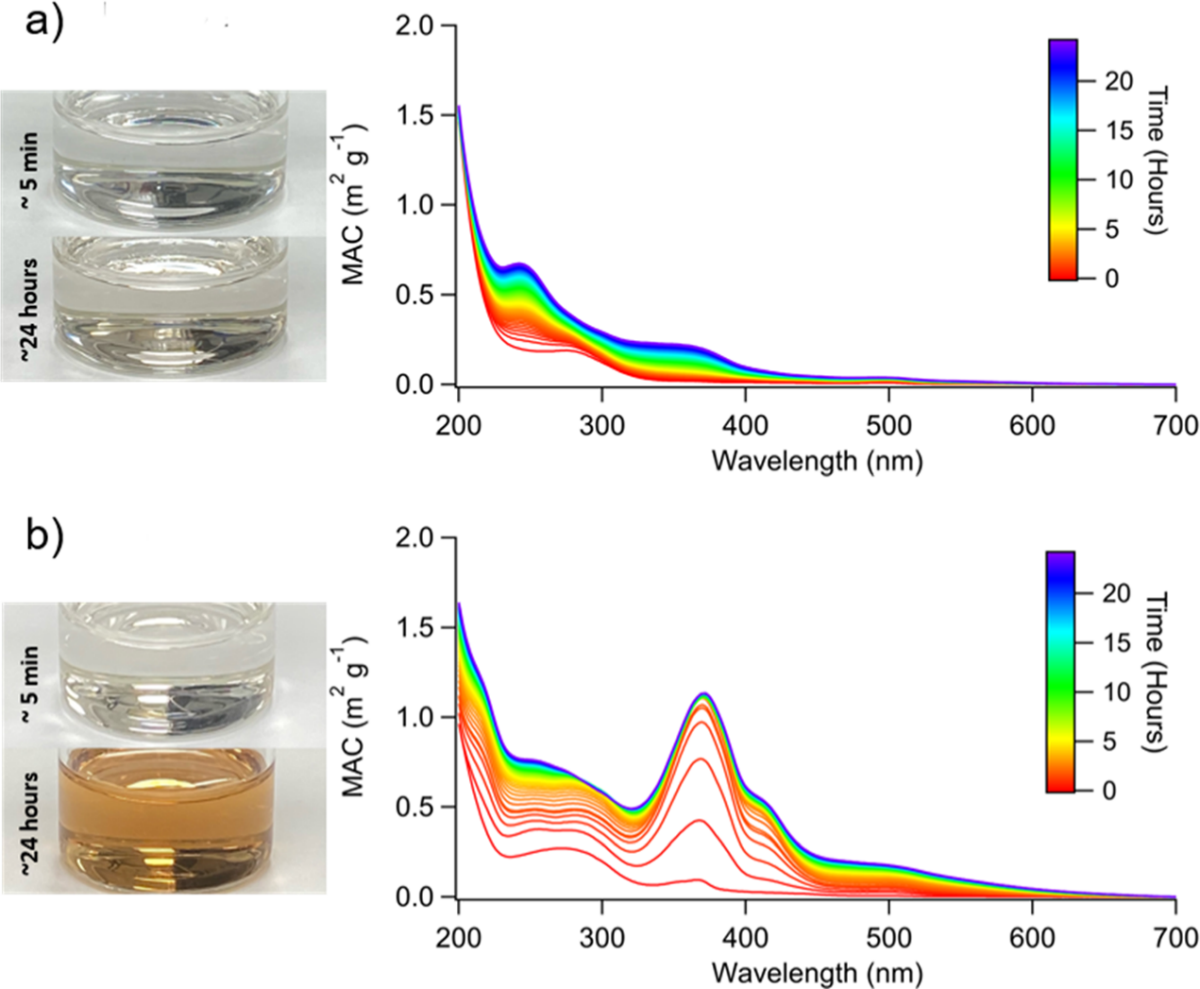
MAC absorption spectra of α-pinene ozonolysis SOA samples aged in (a) 5.6 M (pH −0.86) and (b) 10 M (pH −1.08) of H_2_SO_4_ collected every 15 min over 24 h. Photographs of SOA and acid solution after ∼5 min and ∼24 h of aging.

Figure 3a shows the UV–vis spectra of α-pinene ozonolysis SOA samples aged in 5.6 M H_2_SO_4_ (pH −0.86) taken with a higher time resolution, where each spectrum was collected every 15 min over 24 h. Again, two dominant peaks at 239 and 351 nm were observed. Assuming first-order kinetics, the lifetimes of browning for the peaks of interest were relatively slow, as indicated in Table 3. The time series plots for each peak, in which the lifetimes of browning were calculated, are shown in Figure S4. The color change after 24 h of aging for the pH −0.86 sample is relatively small (see the photograph in Figure 3a). On the other hand, there is a large color change in the α-pinene ozonolysis SOA samples aged at pH −1.08, where after 24 h of aging, the sample has a dark orange, brown tint (Figure 3b). The spectra of SOA samples aged at pH −1.08 (Figure 3b) show five distinct absorption peaks at 254, 273, 370, 418, and 500 nm, all of which have a much faster lifetime of browning (Table 3) than that for the pH −1.08 sample. These observations of the formation of light-absorbing compounds for α-pinene ozonolysis SOA samples aged at pH −0.86 and pH −1.08 are consistent with the change in chemical composition indicated by the respective mass spectra.

**Table 3:** Lifetime of Browning for Several Peaks of Interest in α-Pinene Ozonolysis SOA Samples Aged in 5.6 M (pH −0.86) and 10 M (pH −1.08) H_2_SO_4_^a^

sample	peaks of interest (nm)	lifetime of browning (h)
pH −0.86	239	13 ± 2
	351	49 ± 3
pH −1.08	254	4.6 ± 0.6
	273	3.8 ± 0.6
	370	0.68 ± 0.06
	418	1.51 ± 0.07
	500	5.0 ± 0.3

a Lifetimes were calculated by assuming pseudo-first-order reactions in the time series fits for each peak.

SOA samples aged at pH −1.08 were also analyzed using fluorescence spectroscopy (Figure S5). A fluorescence band appeared at λ_ex_ ≈ 450 nm/λ_em_ ≈ 520 nm. The presence of this band is indicative of the formation of strongly conjugated products.

### UPLC Analysis of the Chromophores

**Figure 4:**
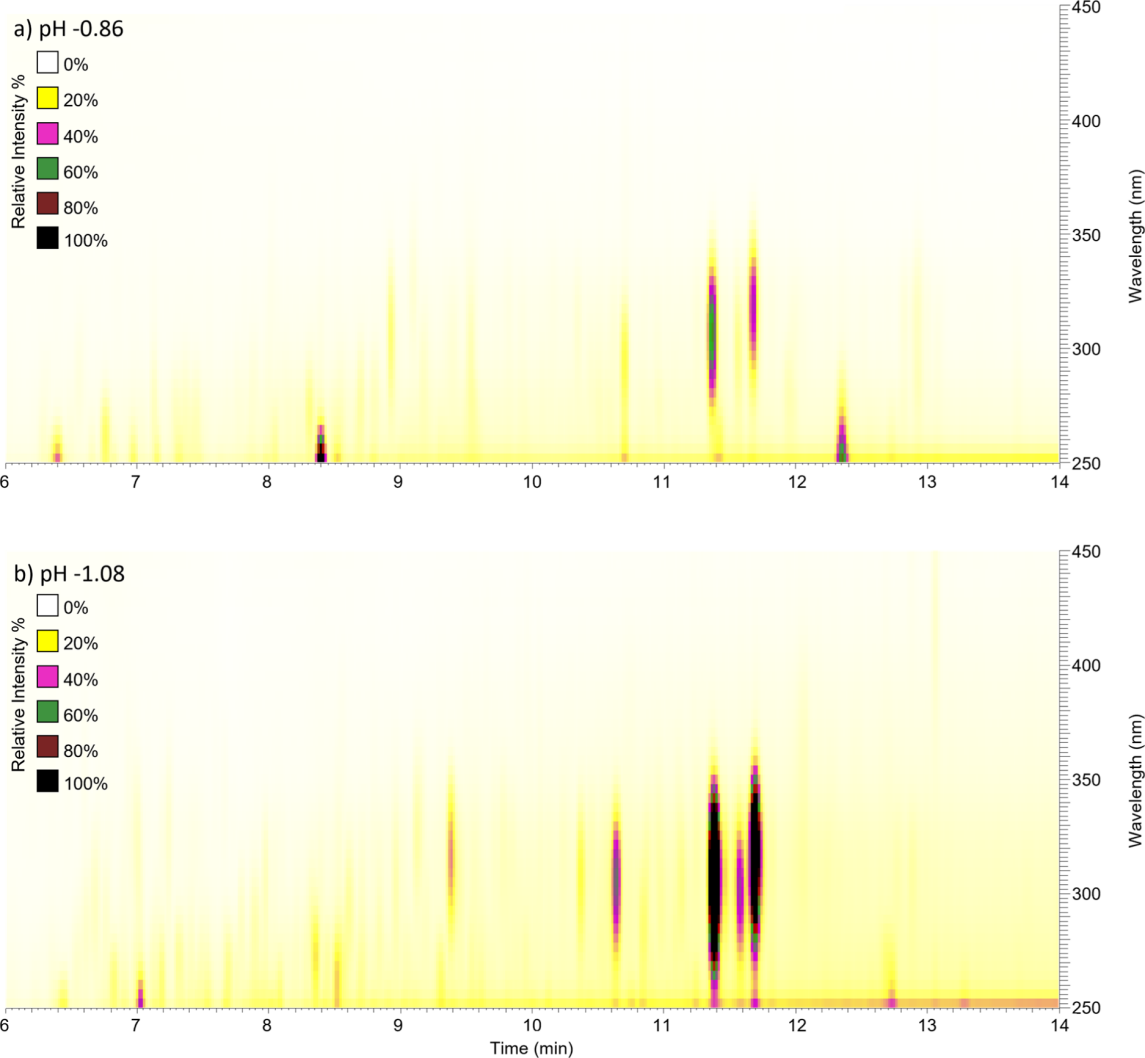
UPLC–PDA chromatograms of α-pinene ozonolysis SOA samples aged in (a) 5.6 M (pH −0.86) and (b) 10 M (pH −1.08) of H_2_SO_4_.

Figure 4 shows the UPLC–PDA chromatograms for α-pinene ozonolysis SOA samples aged in (a) 5.6 M (pH −0.86) and (b) 10 M (pH −1.08) solutions of H_2_SO_4_. The chromatograms have well-defined peaks, which we attempted to identify by correlating the PDA and TIC chromatograms. Due to a very large number of co-eluting compounds, it was difficult to definitively associate the molecular formulas in the HRMS chromatograms with the peaks appearing in the PDA chromatograms. Therefore, additional SOA samples were prepared and aged, and the positive ion mode data were acquired. Figure S6 shows the UPLC chromatograms associated with the PDA and positive ion mode HRMS for α-pinene ozonolysis SOA samples aged in 10 M (pH −1.08) of H_2_SO_4_. The peak at 11.39 min in the PDA chromatogram corresponds to the peak at 11.46 min in the MS chromatogram (there is a 0.06 min time delay between the PDA and the HRMS analyzers). The best match was with the ion at *m*/*z* 151.112 (C_10_H_15_O^+^), which had a strikingly similar single-ion chromatogram to the PDA chromatogram (triplet peaks at 11.39, 11.59, and 11.71 min). Efficient chromophores tend to have either a heteroatom or a high level of aromaticity. However, the C_10_H_14_O compound corresponding to this ion does not have a heteroatom and has a low aromaticity index^73^ of 0.33, suggesting that this may have been a fragment of the original chromophore rather than the chromophore itself. Other co-eluting ions do not have similar chromatograms as the PDA, and higher weight oligomer MS/MS spectra do not have *m*/*z* 151.112 (C_10_H_15_O^+^) as a corresponding fragment. At this time, we do not have conclusive information about the molecular formulas of the chromophores in the aged SOA, but we are currently testing selected α-pinene oxidation compounds (such as pinonic acid and pinonaldehyde) to obtain more information.

### Comparison of PDA and UV–Vis Absorption Spectra

Figure 4a showing the PDA chromatograms indicates that there are four major chromophores that absorb radiation in the near-UV region, with the longest wavelength peaks appearing at 300–350 nm when SOA is aged in 5.6 M H_2_SO_4_. As the concentration of the acid increases to 10 M, a few additional peaks appear; however, all of them are still confined to wavelengths below 350 nm, in stark contrast with the results of Figure 3, which shows measurable absorbance extending beyond 600 nm.

**Figure 5:**
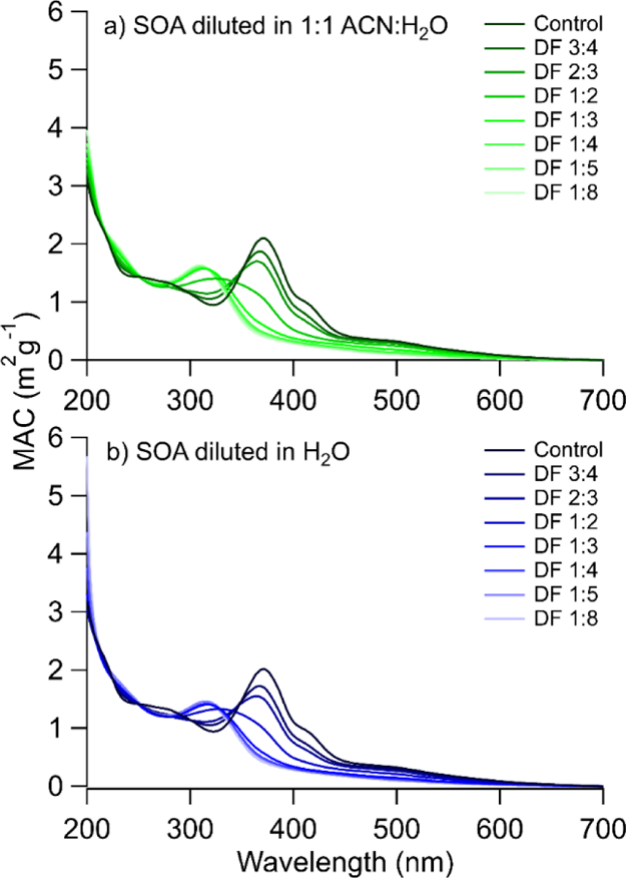
MAC absorption spectra of α-pinene ozonolysis SOA samples aged in 10 M (pH −1.08) of H_2_SO_4_ diluted with 1:1 ACN/H_2_O and H_2_O at various dilution factors. DF, dilution factor.

Initially, we could not reconcile the peak wavelengths observed in the PDA absorption spectra (Figure 4) and UV–vis absorption spectra (Figure 3). For example, no absorption was detected by the PDA above 400 nm, even though MAC of the pH −1.08 sample extended all the way to 700 nm in Figure 3. After verifying the wavelength calibration of the PDA detector, a series of additional experiments were conducted to understand whether this shift was due to the lower acidity of the water–acetonitrile eluent used in UPLC. A sample of SOA aged in acid for 2 days was diluted with the solvents used in the liquid chromatography method, which is outlined in Table S1. The absorption spectra were collected for each sample to monitor the shift in peak absorbance, which are shown in Figure 5.

The sample was first diluted with 1:1 ACN/H_2_O, as shown in Figure 5a, because the majority of the mobile phase constitutes of a mixture of ACN and H_2_O. At a certain level of dilution, the original dominant peak at 354 nm in the spectrum shifts to 310 nm, which corresponds well to the peak seen in the PDA data. The peaks at 418 and 500 nm are also reduced. This explains why we could not detect peaks at longer wavelengths in the PDA data. Using water instead of the water–acetonitrile mixture led to similar observations (Figure 5b). This type of spectral shift is most likely promoted by changing the acid–base equilibria resulting from the sample dilution. There are known precedents for the absorption spectra of atmospherically relevant compounds to be pH-dependent, for example, the spectra of nitrophenols shift to longer wavelengths at basic pH due to the formation of phenolates.^74−76^ There have been other studies that show that the absorption properties of imidazole-2-carboxaldehyde and pyruvic acid can be altered depending on the pH on its environment, which is prompted by acid base equilibria.^77,78^ Additionally, the pH dependence of aerosol absorption has also been detected in field samples collected in southeastern United States and Beijing.^79,80^ It should also be recognized that a large number of acid–base indicators change their spectra at well-defined pH points. It appears that the (currently unidentified) chromophoric products produced from α-pinene SOA in the presence of concentrated sulfuric acid have such similar halochromic properties. Understanding the characteristics of such halochromic chromophores is important, as water in the atmosphere, in the form of water vapor, cloud and fog droplets, and aerosol liquid water, can influence the acidic environment over a wide range.

## Conclusions

Acid-catalyzed and acid-driven reactions can have a large effect on the chemical composition and properties of organic aerosols, which can spend days to weeks in the atmosphere. The impacts of highly acidic conditions on the aerosol chemical composition and optical properties were explored by generating α-pinene ozonolysis SOA and aging the resulting SOA in bulk sulfuric acid solutions with atmospherically relevant acidities. We found that aging of SOA under highly acidic conditions resulted in significant changes in the SOA chemical composition, including the formation of organosulfur compounds and chromophores.

These findings may be especially important in the context of the upper troposphere and the lower stratosphere (UTLS). Aerosols are widespread at high altitudes, most likely formed by the condensation of gas-phase precursors brought up by deep convection.^81−83^ The aerosols in this region are primarily composed of sulfuric acid (40–80 wt %), but they also contain significant amounts of organic compounds.^40,84−86^ The findings from this work further our understanding of how the chemical reactions between sulfuric acid and organic compounds can proceed over the long lifetime of the UTLS aerosols. Specifically, these interactions can change the chemical composition and optical properties of the UTLS aerosols over a long timescale and therefore have a large influence on radiative energy fluxes. However, in this work, the experiments were performed at ambient temperatures, and it is expected that lower temperatures, such as those in the UTLS, can affect the rate of the acid-catalyzed browning processes.

## Supporting Information Available

Graphical summary of aging experiments; mass spectra of fresh α-pinene ozonolysis SOA integrated over the retention time; graph showing the overall amounts of CHO and CHOS compounds in the aged SOA; time-dependent MAC values for peaks of interest; excitation–emission fluorescence spectrum of SOA; positive ion mode HRMS and PDA chromatogram between 3 and 15 min for α-pinene ozonolysis SOA aged at pH −1.08 for 2 days; and solutions used in the peak shift experiments (PDF)
