# Supplementary material for: Highly Acidic Conditions Drastically Alter the Chemical Composition and Absorption Coefficient of α-Pinene Secondary Organic Aerosol
Source: ACS Earth Space Chem. 2022 Nov 22;6(12):2983–94. doi: 10.1021/acsearthspacechem.2c00249 (PMC9762236; doi:10.1021/acsearthspacechem.2c00249)
Supplement: Supplementary file 1 — sp2c00249_si_001.pdf [file sp2c00249_si_001.pdf]

Supporting Information Section

Highly acidic conditions drastically alter chemical composition and absorption coefficient of α-pinene secondary organic aerosol

Cynthia Wong, Sijia Liu, and Sergey A. Nizkorodov

Department of Chemistry, University of California, Irvine, Irvine, CA 92697-2025

Contents

Figure S1: Aging Experiments Summary 2

Figure S2. Retention time integrated mass spectra of fresh α-pinene ozonolysis SOA. 2

Figure S3: Overall Amounts of CHO and CHOS Compounds 3

Figure S4: Time Dependent MAC for peaks of interest in SOA samples aged in pH -0.86 and pH -1.08 3

Figure S5: Fluorescence of APIN SOA 4

Table S1: Solution Acidity in Peak Shift Experiments 5

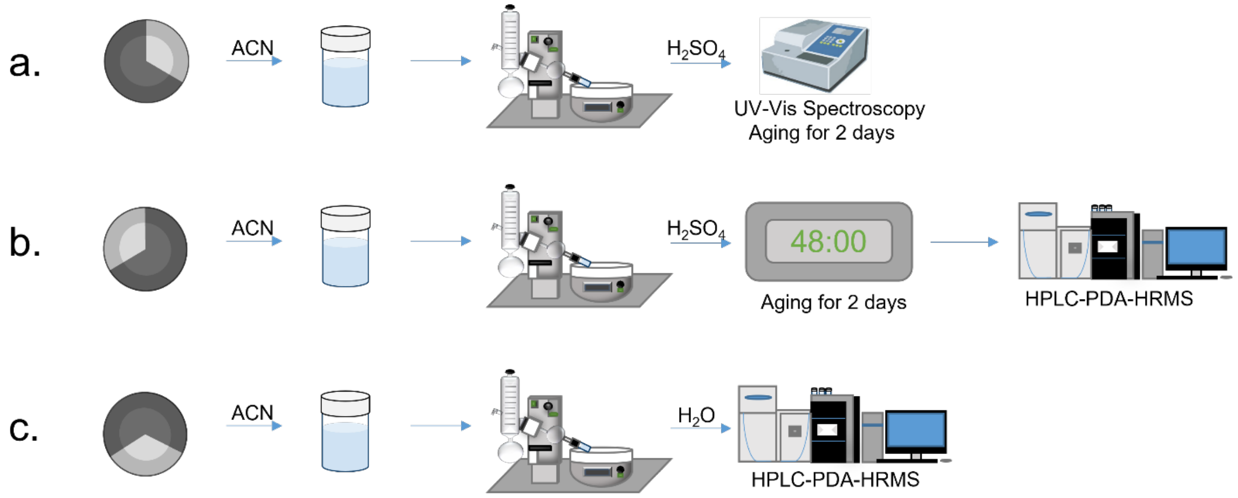

Figure S1: Aging Experiments Summary

SOA were first generated in a flow tube reactor and collected on a foil substrate. Foil substrates were cut into three segments, extracted using acetonitrile, and acetonitrile was removed using a rotary evaporator. The corresponding acid was added to Sample A and Sample B. Sample C was extracted using the same method and dissolved in water for our control group.

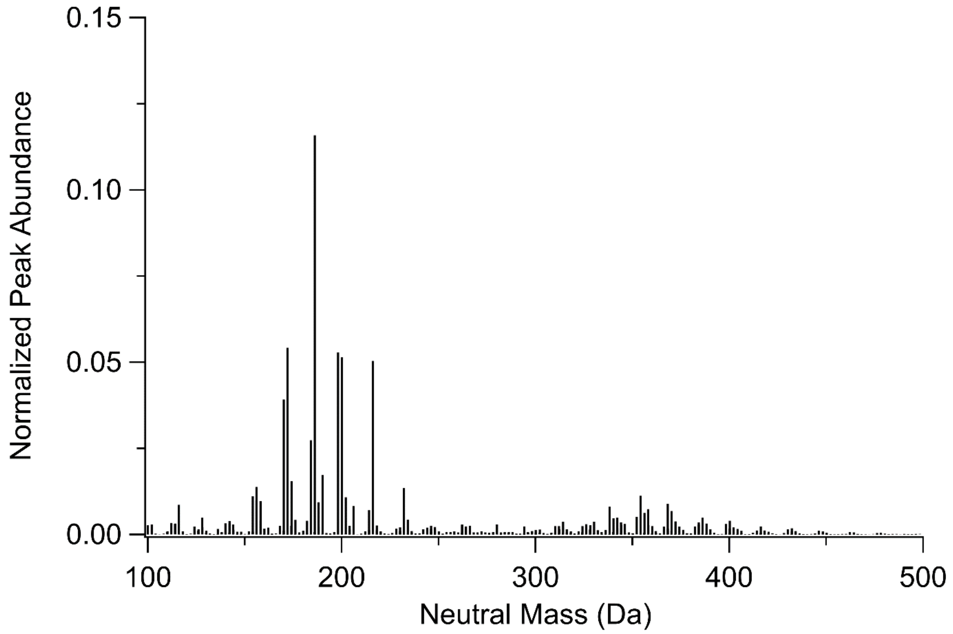

Figure S2. Retention time integrated mass spectra of fresh α-pinene ozonolysis SOA.

Peaks were normalized to the combined peak abundance

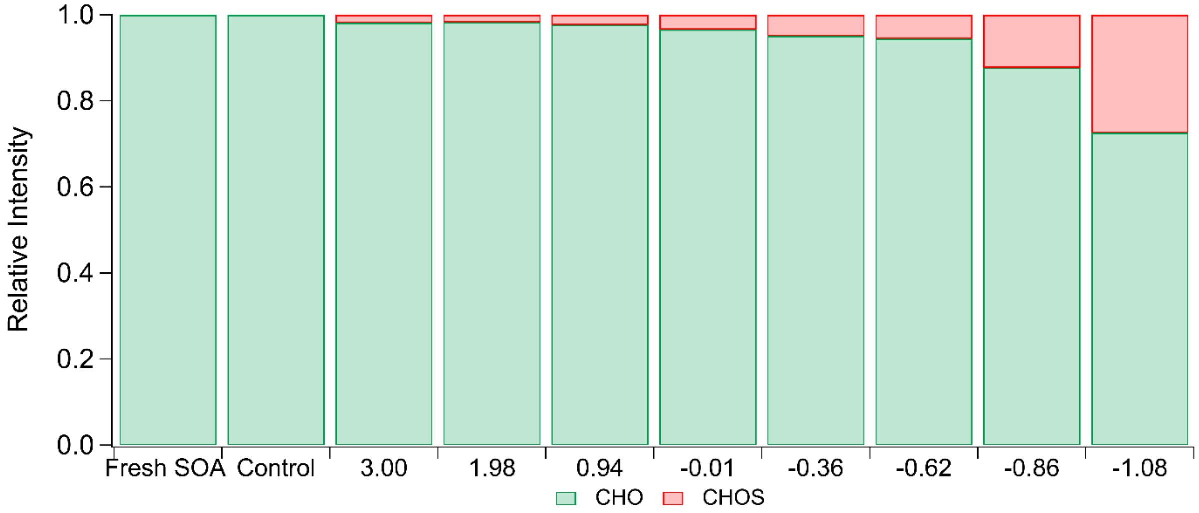

Figure S3: Overall Amounts of CHO and CHOS Compounds

Relative ion abundance of the CHO (green) and CHOS (red) compounds present in fresh and aged α-pinene ozonolysis SOA samples. The labels on the x-axis represent pH values from the E-AIM model (Table 1), except for pH 4.3 sample, which corresponds to the pH meter reading. The ion peak abundances for all observed CHO and CHOS compounds were added. CHOS compounds may be overestimated in this approach as they have higher ionization efficiencies in ESI.

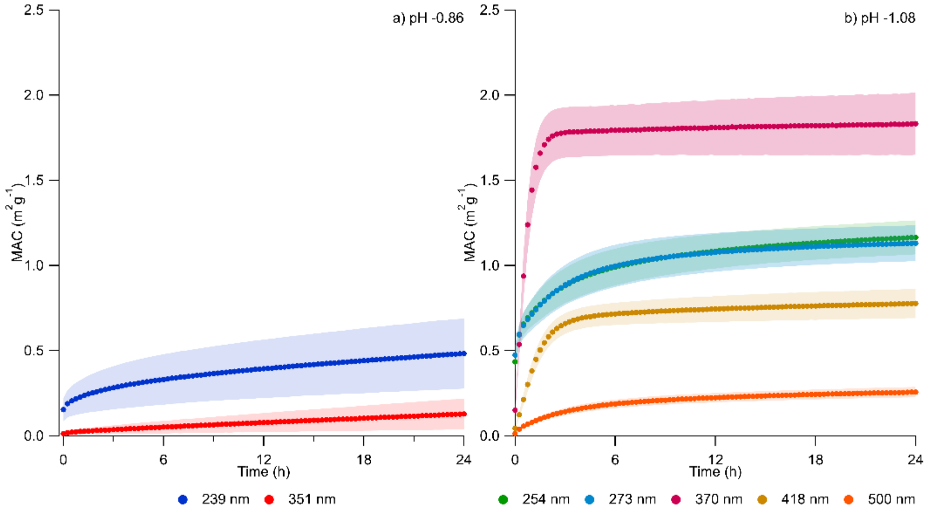

Figure S4: Time Dependent MAC for peaks of interest in SOA samples aged in pH -0.86 and pH -1.08

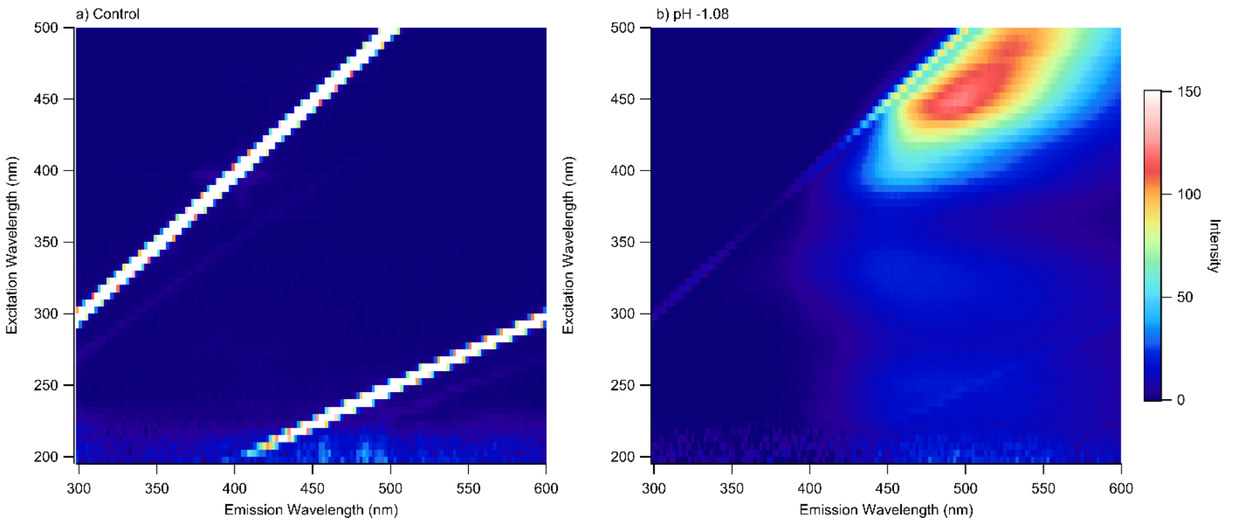

Figure S5: Fluorescence of APIN SOA

Excitation-emission matrix plot for water (a), pH -1.08 solution (b), and SOA aged at pH -1.08 (c). There is no visible fluorescence in the controls (Fig. S5a-b), however a relatively weak fluorescence band appeared at λ_{ex}≈450 nm/λ_{em}≈520 nm in the SOA sample (Fig. S5c).

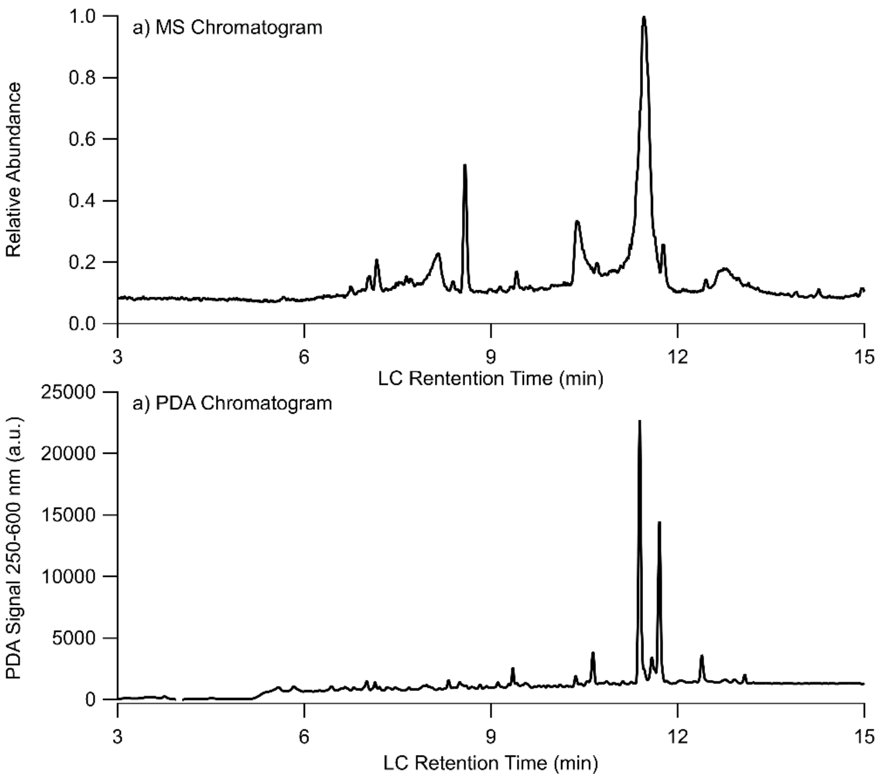

Figure S6: Positive ion mode HRMS and PDA chromatogram between 3-15 min for α-pinene ozonolysis SOA aged in pH -1.08 conditions for 2 days

Table S1: Solution Acidity in Peak Shift Experiments

SOA samples aged at pH -1 were diluted with 1:1 ACN:H_2O and H_2O. Estimated pH was calculated using Extended Aerosol Inorganics Model (E-AIM (http://www.aim.env.uea.ac.uk/aim/aim.php).

**Sample**	**Dilution Factor**	**Estimated pH (E-AIM)**
Control	1	-1.08
3:4 Dilution	1.3	-0.97
2:3 Dilution	1.5	-0.93
1:2 Dilution	2	-0.817
1:3 Dilution	3	-0.646
1:4 Dilution	4	-0.517
1:5 Dilution	5	-0.415
1:8 Dilution	8	-0.2
